# Open-Minded Midwifes, Literate Butchers, and Greedy Hooligans—The Independent Contributions of Stereotype Valence and Consistency on Evaluative Judgments

**DOI:** 10.3389/fpsyg.2017.01723

**Published:** 2017-10-09

**Authors:** Lisa Schubert, Anita Körner, Berit Lindau, Fritz Strack, Sascha Topolinski

**Affiliations:** ^1^Institute of Psychology, University of Würzburg, Würzburg, Germany; ^2^Social Cognition Center Cologne, University of Cologne, Cologne, Germany

**Keywords:** attitudes, stereotype disconfirmation, fluency, consistency, affect

## Abstract

Do people evaluate an open-minded midwife less positively than a caring midwife? Both *open-minded* and *caring* are generally seen as positive attributes. However, consistency varies—the attribute *caring* is consistent with the midwife stereotype while *open-minded* is not. In general, both stimulus valence and consistency can influence evaluations. Six experiments investigated the respective influence of valence and consistency on evaluative judgments in the domain of stereotyping. In an impression formation paradigm, valence and consistency of stereotypic information about target persons were manipulated orthogonally and spontaneous evaluations of these target persons were measured. Valence reliably influenced evaluations. However, for strongly valenced stereotypes, no effect of consistency was observed. Parameters possibly preventing the occurrence of consistency effects were ruled out, specifically, valence of inconsistent attributes, processing priority of category information, and impression formation instructions. However, consistency had subtle effects on evaluative judgments if the information about a target person was not strongly valenced and experimental conditions were optimal. Concluding, in principle, both stereotype valence and consistency can play a role in evaluative judgments of stereotypic target persons. However, the more subtle influence of consistency does not seem to substantially influence evaluations of stereotyped target persons. Implications for fluency research and stereotype disconfirmation are discussed.

## Introduction

When interacting with people, we have immediate spontaneous likes or dislikes. Imagine encountering a hooligan in the street. Most of us would immediately dislike this person because of the valence of the hooligan stereotype. However, imagine a *sweet hooligan*, for instance, a hooligan tenderly soothing his daughter. In this case, something is odd and confusing, and the inconsistency of available information about this target triggers a cognitive feeling of unease and confusion. Would being child-loving render the hooligan even more negative due to the confusion it triggers or more positive due to the positive information it contains? And what about an open-minded midwife compared to a caring midwife? Here, both attributes (open-minded and caring) are positive. However, one is inconsistent with the midwife stereotype (open-minded) while the other one (caring) is consistent. How does consistency influence preferences when valence is kept constant?

The present investigation examines two independent sources of affect during the encoding of information about a social target, namely the affective valence of the information itself (e.g., Russell, 2003, 2009) and the cognitive feeling while encoding this information (e.g., Schwarz, 1990).

Abundant research has investigated the psychological roots of spontaneous preferences, ranging from mere retrieval of affect from memory (Fazio et al., 1986) over integration of current propositional information (e.g., Anderson, 1962, 1971) to meta-cognitive feelings during processing the target (Schwarz, 1990, 2002, 2012). In the following, we will first review previous evidence on the impact of stimulus valence and stimulus consistency on preferences more generally before integrating more specific approaches regarding stereotype consistency.

### Stimulus Valence as a Source for Preferences

The most obvious source of preferences in evaluative judgments is the valence of the target itself. When encountering valenced targets, evaluations are automatically activated from memory (Fazio et al., 1986). Likewise, judgments of liking can be influenced by stimulus features previously associated with positive or negative events by reactivating this positivity or negativity (Fazio and Olson, 2003). If multiple features of a target are available, their respective evaluations are integrated into an overall evaluation of the target (e.g., Massaro and Friedman, 1990). An approach to explain this integrative process are additive accounts of impression formation (e.g., Anderson, 1962, 1971; Kahneman et al., 1993) stating that the values of each single attribute of a stimulus are averaged to reach a net evaluation. The outcome of this process is positive or negative affect (Russell, 2003, 2009), which is then used as the basis for the evaluative judgment (Schwarz, 1990, 2002, 2012).

### Stimulus Consistency as a Source for Preferences

A more indirect source of preferences is stimulus consistency. Maintaining consistency, or coherence, between various aspects of the world is a fundamental aim of the mind (Singer, 1999; Gawronski and Strack, 2012), and humans have developed a strong sensitivity for the presence or absence of coherence (Antonovsky and Sagy, 1986). Consequently, inconsistency between different pieces of information, or between new information and existing knowledge is aversive. It potentially interferes with effective and unconflicted action (Harmon-Jones et al., 2012), or it disrupts the process of establishing meaning from coherent relations in the external world (see the *meaning maintenance model*, Heine et al., 2006; Proulx and Heine, 2008; Proulx et al., 2010; Randles et al., 2011).

Further supporting evidence of the affective impact of stimulus consistency on preferences stems from research on *processing fluency* (Reber et al., 2004), which is the content-independent efficiency with which a stimulus is processed. Consistency, or coherence, increases processing fluency (Topolinski, 2011). Various experiments have shown that high processing fluency is experienced as hedonically positive (Winkielman et al., 2003; Reber et al., 2004). The sort of fluency that is most closely related to stereotype consistency is semantic, or conceptual, fluency (Whittlesea, 1993), which has been shown to elicit positive affect (Topolinski et al., 2009). For example, Topolinski and Strack (2009b) presented participants with word triads that either shared a common associate (e.g., RABBIT CLOUD CREAM; implying WHITE; *coherent*) or were only random words (e.g., DREAM BALL BOOK; *incoherent*) and asked for participants’ spontaneous preference for these word triads. Although participants were not aware of the hidden semantic coherence, they preferred coherent over incoherent triads (Topolinski and Strack, 2009a,b,c), and even showed subtle smiling while reading coherent (compared to incoherent) triads (Topolinski et al., 2009). Moreover, valence has been shown to influence coherence judgments (Topolinski and Strack, 2009b). Participants were presented with semantically coherent and incoherent word triads and were asked to intuitively judge the triads’ coherence. In addition, independent from actual coherence, the triads’ valence was manipulated by constructing coherent and incoherent word triads out of relatively positive (HOLY LIQUID FRESH; implying WATER) or negative words (SALT RAIN DROWN; also implying WATER), respectively. It was found that not only coherent triads were judged to be coherent more often than incoherent triads, but that positive triads, too, were judged to be coherent more frequently than negative triads. Although this finding pertains to rather neutral non-social word triads, and the dependent measure was coherence intuitions, this evidence shows that valence and consistency, if pit against each other, can show additive effects.

In sum, from both inconsistency-threat (e.g., Heine et al., 2006; Harmon-Jones et al., 2012) and fluency approaches (e.g., Topolinski and Strack, 2009a,b) the prediction can be derived that consistency compared to inconsistency induces positive affect. Importantly, the positive affect resulting from consistency should be independent from actual stimulus content (e.g., its valence, see Topolinski and Strack, 2009b).

### Consistency in Stereotypes: the Effects of Stereotype Disconfirmation

In social psychology, a classical example of striving for consistency is holding and maintaining knowledge about stereotypes (Weber and Crocker, 1983; Devine, 1989; Devine and Sharp, 2009). The present approach examines *stereotype disconfirmation* (e.g., Macrae et al., 1999; Sherman et al., 2012), the exhibition of behaviors or features of a stereotypical target that are inconsistent with the current stereotype.

Generally, stereotype-inconsistent compared to consistent information has been found to receive more attention (Brannon et al., 2017), to be processed more deeply (Fiske, 1980; Bargh and Thein, 1985; Pezzo, 2003), and, as a consequence, to be remembered better (Hastie and Kumar, 1979; Bargh and Thein, 1985; Schützwohl, 1998; for a review, see Stangor and McMillan, 1992; but see, for opposite effects, Fyock and Stangor, 1994; Johnston, 1996; Trope and Thompson, 1997; for an integration, see Sherman et al., 2012). This memory bias is even stronger, when the (counter-)stereotypic information is of special relevance to the perceiver (Süssenbach et al., 2016).

Thus, there is abundant research on the influence of stereotype disconfirmation on memory. However, the effects of stereotype disconfirmation on evaluative judgments has been less extensively examined. Importantly, up to now, there seems to be no research on the influence of consistency on preference for the target person. Previous research found that individuals who described others in stereotype-consistent ways were evaluated more positively than those who described others in stereotype-inconsistent ways (Castelli et al., 2005). The focus of the present research, however, is the question how an (in)consistent target person is evaluated him- or herself. Some experiments on memory biases for stereotype-inconsistent information assessed evaluative judgments for inconsistent targets but not in a way to enable testing differences in preference between consistent and inconstant targets (due to a within-target manipulation of consistency; Förster et al., 2000, 2004; or due to the fact that only inconsistent targets were shown, Förster et al., 2004). Another experiment that assessed evaluations of stereotype-inconsistent target persons was conducted by Bless et al. (2001). German participants read a description of a member of the ethnic group of Sinti and Roma (negative stereotype in Germany). The target person was described rather favorably and therefore inconsistent with that stereotype. However, because this experiments’ design focused on factors other than consistency vs. inconsistency, participants only received inconsistent target persons, so no comparison between consistent and inconsistent target persons was possible.

Furthermore, there is also work on the evaluation of stereotype-inconsistent behavior of target persons. A study by Lambert and Wyer (1990), for instance, had participants read a description of a target person who was labeled as either being a priest (highly moral) or a businessman (moderately moral) and who exhibited an immoral behavior (stealing). Even though the general evaluation of priests was positive, the evaluation of the priest showing immoral behavior was just as negative as the evaluation of a businessman showing immoral behavior (Lambert and Wyer, 1990). Participants’ evaluative judgment of the target person was not influenced by his group membership, but by his individual behavior. However, a comparison between stereotype-consistent and -inconsistent behavior was not possible because participants only evaluated target persons displaying stereotype-inconsistent (immoral) behavior.

One series of experiments actually compared evaluative judgments of stereotype-consistent and -inconsistent target persons (Mendes et al., 2007). Reactions toward typical and atypical members of a social category were compared. Specifically, participants interacted with a confederate who was either a typical or an atypical member of his or her social category (e.g., white vs. black with high vs. low socio-economic status; or Asian with or without southern accent). The result was that stereotype-consistent interaction partners were preferred over inconsistent ones and elicited less physiological threat responses (Mendes et al., 2007). This evidence is in line with the predictions from inconsistency-threat and fluency that inconsistency should elicit more negative evaluations.

### Aim of the Present Work: Effects of Stereotype Valence and Consistency

To our knowledge, up to now there has been no attempt to manipulate stereotype valence and stimulus consistency orthogonally (but see the mixed evidence on stimulus valence and fluency due to repetition, Grush, 1976; Dijksterhuis and Smith, 2002; Schellenberg et al., 2008).

The present research goes beyond earlier approaches by systematically mapping stereotype valence against consistency. Furthermore, consistency itself was manipulated more rigorously. While earlier work (e.g., Förster et al., 2000, 2004; Mendes et al., 2007) used only a single target person and chose the single stereotype-(in)consistent behavior or feature arbitrarily (which allows material effects), the present experiments used multiple targets, each with multiple features, that were participant-generated (see Pilot Studies 1 and 2). Additionally, stereotype-consistent and -inconsistent features were sampled from the same stimulus pool, thus preventing material effects. However, the main additional contribution of the present work is the independent manipulation of valence and consistency, which is outlined in the following.

Concluding from the above reviewed literature on stereotype disconfirmation (e.g., Mendes et al., 2007) and processing fluency (Topolinski and Strack, 2009a,b,c) we predicted that stereotype valence and stereotype consistency would independently influence preferences, with more positive evaluations for positive compared to negative stereotypes, and also for consistent compared to inconsistent targets, with no interactional effects between valence and consistency. The previously reviewed experiment by Topolinski and Strack (2009b), showing that coherence and valence showed additive effects for consistency judgments, can be seen as supporting evidence. The present experiments examined whether this independent contribution also holds for preferences for stereotyped target persons. This research agenda has the theoretical significance of testing whether psychological processes found in basic cognitive research, in this case, the additive principle of valence and semantic coherence, also holds for social perception and person impression formation. Thereby, we explore the underlying information processing steps that drive social behavior and test which role well-established cognitive effects play in a social context.

In Experiment 1, we compared consistent target persons to valence inconsistent as well as semantically inconsistent targets. This experiment addressed the aforementioned question how a sweet hooligan is evaluated compared to a brutal hooligan. In Experiments 2–4, we compared consistent targets to semantically inconsistent targets, but kept valence within a target person constant. For instance, we expected an open-minded midwife to be evaluated more negatively than a caring midwife. In Experiments 5 and 6, we examined neutral stereotypes and again compared semantically consistent with inconsistent targets. We expected a literate butcher to be more negatively evaluated than a strong butcher. Thus, in all three cases we expected that consistency compared to inconsistency would increase fluency-induced positive affect, which should, in turn, lead to more favorable evaluations.

### Data Treatment

We determined the required sample sizes with a power analysis (*G^∗^Power 3*, Faul et al., 2007), attempting a power of 0.80, an alpha-level of 0.05, and estimating the effect size based on previously published effects of coherence on liking for word triads outside a social context (Topolinski and Strack, 2009a, Experiment 3, p. 1484, *N* = 61, *d_z_* = 0.35; Topolinski and Strack, 2009c, Experiment 1, p. 611, *N* = 22, *d_z_* = 0.84). The pooled effect size was *d_z_* = 0.48, which equals *f* = 0.24. Therefore, we used *f* = 0.24 as an estimate for the expected size of the consistency effect. A power analysis for a repeated-measures ANOVA with these parameters (as well as a correlation of 0.5, and non-sphericity correction of 1) yields *N*_req_ = 37. Because all experiments were combined with other studies, some of which required larger sample sizes, most Experiments exceed the required sample size; only Experiment 4 fell below this criterion. As the observed effect sizes in Experiments 1–4 were much smaller than expected, we drastically increased the sample sizes for the last two experiments to about 200 participants each. Additionally, Experiments 5^1^ and 6^2^ were preregistered.

We report all manipulations and measures. No subjects were excluded from the analyses and all preparatory steps prior to the main analyses are described in the text. All experiments were lab-based and took place at German universities; participants were predominantly students majoring in various disciplines. We used Medialab/Direct RT for data collection.

### Pilot Studies

In two pilot studies, a new pool of stimuli was developed based on semantic and affective associations of participant samples comparable to the participants in the later experiments.

#### Pilot Study 1

This Pilot Study identified social stereotypes that are strongly valenced. Fifty-seven social stereotypes were gathered from various internet sources and by personal communication with other social scientists with the eventual categories being social and occupational (e.g., professor, gardener), recreational (e.g., rock climber, dancer), or other (e.g., criminal, billionaire). *N* = 49 participants (mean age = 24, *SD* = 4; 39 female, 10 male) rated the categories as part of a multi-experiment session, earning course credit or financial compensation.

Participants read one stereotype at a time on a computer screen in random order and were asked to evaluate each category by pressing the according key on the keyboard (*What are your feelings towards …*, scale ranging from 1 = *very negative* to 7 = *very positive*, Tarrant and Hadert, 2010). Mean evaluation was *M* = 4.24 (*SD* = 0.48), ranging from *M* = 1.27 (*SD* = 0.57, blackmailer) to *M* = 6.47 (*SD* = 0.89, volunteer).

According to this distribution of evaluations, the four most positive (*M* > 5), neutral (3 < *M* < 5), and negative (*M* < 3) items were chosen with the only constraint that stereotypes should be sufficiently distinctive as judged by the authors (e.g., blackmailer and criminal were judged to be not sufficiently distinctive). The resulting items were: positive: pilot, midwife, fireman, and volunteer; neutral: racing driver, butcher, detective, and bookbinder (used in Experiments 5 and 6); and negative: blackmailer, hooligan, early school leaver, and insurance salesman (means reported in **Table 1**).

**Table 1:** Standardized set of categories and consistent attributes.

**Positive categories**			
Volunteer	Fireman	Midwife	Pilot
(*Ehrenamtliche*)	(*Feuerwehrmann*)	(*Hebamme*)	(*Pilot*)
*M* = 6.47, *SD* = 0.89	*M* = 6.00, *SD* = 1.37	*M* = 5.96, *SD* = 1.17	*M* = 5.24, *SD* = 1.16
Helpful	Brave	Caring	Confident
(*hilfsbereit*)	(*tapfer*)	(*fürsorglich*)	(*souverän*)
Friendly	Heroic	Child-loving	Intelligent
(*nett*)	(*heldenhaft*)	(*kinderlieb*)	(*intelligent*)
Dedicated	Strong	Affectionate	Educated
(*engagiert*)	(*stark*)	(*herzlich*)	(*gebildet*)
Selfless	Fast	Warm-hearted	Attractive
(*selbstlos*)	(*schnell*)	(*warmherzig*)	(*attraktiv*)
Fair	Assiduous	Empathic	Open-minded
(*gerecht*)	(*gewissenhaft*)	(*einfühlsam*)	(*weltoffen*)
**Neutral categories**			
Book Binder	Detective	Butcher	Racing Driver
(*Buchbinderin*)	(*Detektiv*)	(*Fleischerin*)	(*Rennfahrer*)
*M* = 4.84, *SD* = 1.30	*M* = 4.14, *SD* = 1.43	*M* = 3.63, *SD* = 1.55	*M* = 3.41, *SD* = 1.24
Literate	Clever	Strong	Adventurous
(*belesen*)	(*schlau*)	(*kräftig*)	(*risikofreudig*)
Boring	Nondescript	Rough	Young
(*langweilig*)	(*unscheinbar*)	(*grob*)	(*jung*)
Accurate	Nosy	Plump	Fast
(*sorgfältig*)	(*neugierig*)	(*dick*)	(*schnell*)
Industrious	Brave	Friendly	Athletic
(*fleißig*)	(*mutig*)	(*freundlich*)	(*sportlich*)
Skillful	Precise	Down-to-earth	Competitive
(*geschickt*)	(*gewissenhaft*)	(*bodenständig*)	(*ehrgeizig*)
**Negative categories**			
Blackmailer	Hooligan	Early school leaver	Insurance salesman
(*Erpresserin*)	(*Hooligan*)	(*Schulabbrecherin*)	(*Versicherungsvertreter*)
*M* = 1.27, *SD* = 0.57	*M* = 1.31, *SD* = 0.71	*M* = 2.51, *SD* = 0.91	*M* = 2.57, *SD* = 1.26
Evil	Violent	Lazy	Pushy
(*böse*)	(*gewaltbereit*)	(*faul*)	(*aufdringlich*)
Ruthless	Brutal	Stupid	Sneaking
(*skrupellos*)	(*brutal*)	(*einfältig*)	(*hinterlistig*)
Greedy	Disrespectful	Aimless	Deceitful
(*habgierig*)	(*respektlos*)	(*orientierungslos*)	(*verlogen*)
Unfair	Loud	Dull	Insistent
(ungerecht^b^/feige^c^)	(*laut*)	(*lustlos*)	(*hartnäckig*)
Unfeeling	Hard-drinking	Undisciplined	Selfish
(*kaltherzig*)	(*trinkfest*)	(*undiszipliniert*)	(*egoistisch*)

*Original German stimuli in italics. ^b^Experiments 1–3; ^c^Experiments 4–6.*

#### Pilot Study 2

This Pilot Study generated attributes that are semantically associated with the stereotypes from Pilot Study 1. An independent sample (*N* = 36, mean age = 26, *SD* = 8; 28 female, 7 male, 1 unknown) participated as part of a multi-experiment session, earning course credit or financial compensation.

Participants were asked to list attributes they spontaneously associated with each of the 12 categories. They received the category labels on a computer screen, one at a time in random order and were asked for attributes they associated with each category (*Which ATTRIBUTES do you spontaneously associate with* …). Participants typed in their associations using the computer keyboard. After 40 s, the next trial started automatically.

Depending on the different categories, between 7 (detective) and 18 (bookbinder) different attributes were reported by at least two participants. Very similar attributes were grouped using the most common name. From the resulting pool of associations, five attributes were chosen for each category using the following criteria: Each attribute had to be among the 10 most frequently named attributes for each category and it had to be rated as sufficiently specific for the particular group by the authors (e.g., *nice* is an attribute fitting almost all positive groups and hence was regarded as too unspecific). The resulting attributes can be found in **Table 1**.

Pilot Studies 1 and 2 thus resulted in a standardized set of four negative, four neutral, and four positive social categories with five semantically consistent attributes each.

## Experiment 1

Target exemplars from positive or negative social stereotypes were presented together with attributes that were either evaluatively and stereotypically consistent (e.g., a caring midwife) or partially evaluatively and stereotypically inconsistent (e.g., a brutal midwife). Then, spontaneous preference for these targets was assessed. In the case of positive targets, (negative) valence and (negative) disfluency of inconsistent information would both decrease preference. Crucially, however, in the case of negative targets, information valence and disfluency of inconsistent information have opposite effects, with the valence of inconsistent attributes being positive (e.g., a child-loving hooligan), but the feeling of disfluency being negative (e.g., Topolinski et al., 2009). Thus, the pattern of the respective impact of disfluency on positive and negative targets should allow an estimation of the respective impact of stimulus-inherent valence and consistency-driven fluency.

### Method

#### Main Experiment

##### Participants

Participants were *N* = 50 students (36 female, 14 male) with a mean age of 25 years (*SD* = 5).

##### Materials

The four positive and four negative stereotypes together with their attributes developed in Pilot Studies 1 and 2 were used (see **Table 1**) and were presented as target persons with a first name (gender was counter-balanced across conditions). Consistent targets were the stereotypes with their respective five attributes (e.g., *Andreas is a Hooligan. He is… brutal, loud, violent, hard drinking, rude*). Inconsistent targets were construed by replacing one attribute (the third, fourth, or fifth attribute that was presented, re-randomized anew for each participant) by a randomly chosen (cf., Hastie and Kumar, 1979) attribute from the opposite-valence categories (e.g., *Andreas is a Hooligan. He is… brutal, loud, violent, caring, rude*; with CARING used from the stereotype *midwife*). As participants saw only one exemplar per stereotype, consistency was balanced across two versions of the material. Thus, each participant evaluated eight targets, with two targets per condition (positive consistent, positive inconsistent, negative consistent, negative inconsistent).

##### Procedure

Participants were informed that they would be presented with information about different persons. They were asked to form a personal impression about these target persons. In each trial, the first name, stereotype, and the five attributes were presented until participants pressed a key to signal that they had read the information (self-paced). Then, the information disappeared, and participants were asked for their evaluation of the current target person by pressing the according number key on the PC keyboard (*What are your feelings towards …*, 1 = *very negative*, 7 = *very positive*, cf., Tarrant and Hadert, 2010). Then, the next target person was presented. The eight target persons (two negative consistent, two negative inconsistent, two positive consistent, two positive inconsistent) were presented in random order, re-randomized anew for each participant. The task took about 3 min and was part of a multi-experiment session.

### Results

**FIGURE 1:**
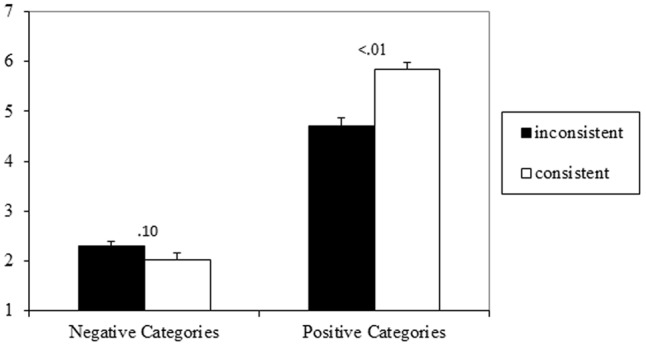
Means and standard errors for evaluation of target persons in Experiment 1 (1 = very negative, 7 = very positive).

Evaluations of targets in the same category were averaged and then entered into a 2 (valence: positive, negative) × 2 (consistency: consistent, inconsistent) repeated measures ANOVA. A main effect of valence emerged, *F*(1,49) = 525.48, *p* < 0.001, $\eta_{p}^{2}$ = 0.92, 90% CI = 0.87–0.93, indicating that positive stereotypes (*M* = 5.28, *SD* = 0.73) were evaluated more positively than negative stereotypes (*M* = 2.16, *SD* = 0.61). Additionally, a main effect of consistency emerged, *F*(1,49) = 14.11, *p* < 0.001, $\eta_{p}^{2}$ = 0.22, 90% CI = 0.07–0.37, indicating that consistent targets (*M* = 3.94, *SD* = 0.62), were evaluated more positively compared to inconsistent targets (*M* = 3.50, *SD* = 0.63). However, this main effect was qualified by an interaction between consistency and valence, *F*(1,49) = 35.34, *p* < 0.001, $\eta_{p}^{2}$ = 0.42, 90% CI = 0.24–0.55. Simple comparisons found that for positive stereotypes, consistent targets (*M* = 5.85, *SD* = 0.83) were evaluated more positively than inconsistent targets (*M* = 4.71, *SD* = 1.06), *t*(49) = 6.63, *p* < 0.001, *d_z_* = 0.94, 95% CI = 0.60–1.27. However, for negative stereotypes, consistent targets (*M* = 2.02, *SD* = 0.90) tended to be rated as less positive than inconsistent targets (*M* = 2.29, *SD* = 0.74), *t*(49) = -1.70, *p* = 0.10, *d_z_* = 0.24, 95% CI = -0.04 to 0.52. Results are illustrated in **Figure 1**.

### Discussion

Experiment 1 found a main effect of stereotype valence, which is a rather trivial finding. While there also was a main effect of consistency, this effect is due to the larger difference in ratings of positive compared to negative targets, as can be seen in **Figure 1**. This particular impact of valence-inconsistent information on positive stereotypes (e.g., the brutal midwife) can be interpreted as a classic negativity bias (Rozin and Royzman, 2001) or a contrast effect due to shifting standards (Biernat and Manis, 1994; Biernat et al., 1998).

The significant interaction effect rather suggests that the net evaluation of the target persons simply depended on the total amount of positive or negative information provided, which is in line with additive accounts of information integration (e.g., Anderson, 1962, 1971): the higher the number of positive attributes ascribed to a target person, the more positive the evaluation of the target person.

The fact that consistency did not influence judgments positively is intriguing because an extensive body of research has shown effects of consistency on preference (e.g., Topolinski and Strack, 2009b; Winkielman et al., 2012). This can be due to at least two factors. First, the affect elicited by the strong stereotype valence of the present stimulus pool (cf., Fazio and Olson, 2003) might be much stronger than the affect elicited by consistency-driven fluency (cf., Russell, 2003), since fluency-driven affective variations are very subtle in nature (Winkielman and Cacioppo, 2001; Topolinski et al., 2009). The affect feeding into the evaluative judgment would then be driven by the stronger affect elicited by stimulus valence, overriding the weaker affect elicited by consistency-driven fluency. This issue will eventually be addressed in Experiments 5 and 6.

Secondly, in the present set-up, the consistency manipulation itself was confounded with valence, because the inconsistent attribute was selected from opposite-valence stereotypes. Thus, the inconsistent attribute was not only semantically inconsistent with the given stereotype, but also evaluatively. In the critical case of a negative stereotype with an inconsistent (positive) attribute, these two forms of inconsistency (semantic and affective) work against each other. The negative affect that should be elicited by the inconsistency-driven lack of processing fluency is countered by the positive affect elicited by positive valence of the attribute itself.

To address this latter issue, Experiment 2 used a semantic consistency manipulation free from confounding affective consistency, by using inconsistent attributes of the same valence. For example, *lazy* is not part of the hooligan stereotype, but still negative. Thus, we kept the net target valence constant (both the stereotype hooligan and the inconsistent attribute lazy are negative). This more subtle affective variations induced by mere semantic consistency might be more likely to feed into preference judgments, thus making an effect of consistency-driven fluency more likely to emerge.

## Experiment 2

This experiment investigated the impact of stereotype valence and stereotype consistency on preferences by manipulating *semantic* consistency (*conceptual fluency*, Whittlesea, 1993) while keeping affective matching (cf., Klauer and Musch, 2002; Topolinski and Deutsch, 2013) constant. Therefore, in the crucial inconsistent conditions, the target persons were presented with valence-consistent but semantically inconsistent attributes (e.g., *athletic midwife, lazy hooligan*).

To increase the likelihood that inconsistent target persons actually were perceived as inconsistent, the number of inconsistent attributes was increased from 1 to 3 out of 5 (e.g., a *hooligan* that is *violent, brutal, lazy, presumptuous*, and *greedy*; with the last three attributes being semantically unrelated to the stereotype (see Pilot Study 2). Thus, the average stimulus valence of attributes presented with the target person was the same for consistent and inconsistent target persons and only varied as a function of stereotype valence. The affect elicited by processing fluency, however, should vary as a function of consistency, with inconsistent attributes being less fluent to process and thus triggering brief negative affect (Topolinski et al., 2009; Topolinski and Strack, 2009a,b). This should lead to a less positive evaluation of inconsistent compared to consistent target persons, irrespective of a main effect of stereotype valence.

To further ensure that consistency is rigorously manipulated and thus likely to have an effect, we conducted a manipulation check in addition to the main experiment, on an independent sample that assessed whether (in)consistency as manipulated in the present case was actually experienced.

### Method

#### Participants

Participants were *N* = 44 students (38 female, 6 male) with a mean age of 26 years (*SD* = 6).

#### Materials and Procedure

Experiment 2 was similar to Experiment 1 apart from the following changes. First, the number of inconsistent attributes in the inconsistency conditions was increased to 3 out of 5, with the first two attributes always being consistent with the current stereotype. Second, the semantically inconsistent attributes for positive (negative) stereotypes were randomly sampled (re-randomized anew for each participant) from the attributes consistent with one of the other three positive (negative) stereotypes from the stimulus pool (e.g., *open-minded midwife*, with open-minded stemming from the positive stereotype pilot; *lazy hooligan* with lazy stemming from the negative stereotype early school leaver). Again, (in)consistency assignment was counter-balanced across participants so that no stereotype would repeatedly appear for a given participant. The dependent variable and the procedure were identical to Experiment 1.

#### Manipulation Check of the Consistency Manipulation

For *N* = 29 independent participants (22 female, 5 male, 2 unknown, mean age 27 years, *SD* = 8) a rigorous manipulation check of coherence was conducted. These participants received the attributes of each condition (positive consistent, positive inconsistent, negative consistent, negative inconsistent) without the target’s ostensible first names and without the stereotype label (see also Experiments 3 and 4), to assess the experienced mere coherence of (in)consistent attributes themselves (cf., Topolinski and Strack, 2009b,d). Instead of reporting their preference, participants were asked to report how coherent the group of words seemed to them (*How coherent does this group of words seem to you …*, 1 = *completely random*, 7 = *very coherent*, cf., Topolinski and Strack, 2009b).

A 2 (valence: positive, negative) × 2 (consistency: consistent, inconsistent) repeated measures ANOVA on the coherence judgments found a main effect of valence, *F*(1,28) = 8.67, *p* < 0.001, $\eta_{p}^{2}$ = 0.24, 90% CI = 0.04–0.42, with positive attribute sets being judged to be more coherent than negative attribute sets (replicating a valence-coherence spill-over already shown by Topolinski and Strack, 2009b, Experiments 4–11). This result can be explained by the higher density of positive information (Unkelbach et al., 2008), meaning that positive stimuli are generally perceived as more similar to one another than negative stimuli. Much more importantly, there was also a main effect of consistency, *F*(1,28) = 40.93, *p* < 0.001, $\eta_{p}^{2}$ = 0.59, 90% CI = 0.37–0.71, with consistent sets of attributes being rated as more coherent than inconsistent sets. There was no interaction between valence and consistency (*F* < 1). This strongly suggests that semantic consistency was effectively manipulated in the current stimulus set.

### Results

**FIGURE 2:**
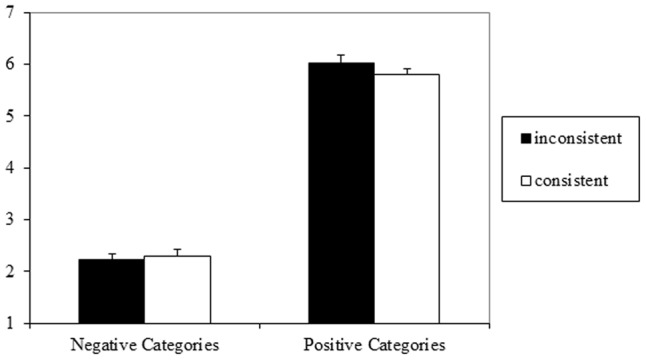
Means and standard errors for evaluation of target persons in Experiment 2 (1 = very negative, 7 = very positive).

The 2 (valence: positive, negative) × 2 (consistency: consistent, inconsistent) repeated measures ANOVA on the preference judgments yielded a main effect of valence, with more positive evaluations of target persons from positive (*M* = 5.91, *SD* = 0.83) compared to negative stereotypes (*M* = 2.23, *SD* = 0.71), *F*(1,43) = 344.10, *p* < 0.001, $\eta_{p}^{2}$ = 0.89, 90% CI = 0.83–0.92. Consistency had no effect, *F*(1,43) = 0.59, *p* = 0.45, $\eta_{p}^{2}$ = 0.01. There was a tendency toward an interaction between valence and consistency, *F*(1,43) = 2.74, *p* = 0.11, $\eta_{p}^{2}$ = 0.06; however, simple comparisons showed no significant differences [positive valence: *t*(43) = 1.64, *p* = 0.11; negative valence, *t*(43) = 0.47, *p* = 0.64]. Findings are illustrated in **Figure 2**.

### Discussion

Consistency did not influence preference judgments for valenced stereotypical target persons. This was the case even though the present purely semantic consistency manipulation was designed completely independent from affective matching (in contrast to Experiment 1), and involved a larger number of inconsistent attributes in the inconsistent condition (three of five instead of one of five attributes in Experiment 1). Moreover, the manipulation check (using an independent sample of participants) confirmed that the present attribute sets effectively manipulated coherence.

Apart from the possibility already mentioned in Experiment 1, that stimulus valence constitutes a stronger cue than consistency and therefore solely influences participants’ evaluations, another possible explanation is that the presented stereotype label simply overshadowed the information given by the attributes. Specifically, it is not clear whether the current evaluative judgments draw on the valence of the stereotype label or on the net valence of the attributes, or both. In terms of the continuum model by Fiske and colleagues (Fiske and Neuberg, 1990; Fiske et al., 1999; Fiske, 2012), the (in)consistent attributes are individuating information, and inconsistency should have motivated piecemeal processing of the single attributes in inconsistent trials. In consistent trials, participants should have relied on category information when making their evaluative judgment (Fiske, 2012). The lacking impact of coherence might suggest that inconsistency was not sufficiently motivating participants to process individuating information but to rather rely on the provided stereotype label. To investigate this possibility, Experiment 3 changed the experimental set-up. Targets were presented without stereotype labels, thus forcing participants to use individuating information in forming their judgments.

## Experiment 3

The presence of social category labels might have influenced evaluations in Experiments 1–2. Social category membership is commonly used in evaluative judgments of others (e.g., Fiske and Neuberg, 1990; Ford et al., 1994; Fiske et al., 1999; Fiske, 2012) and merely perceiving a category label has been found to activate stereotypically associated traits and attributes (Devine, 1989). Also, assigning a target person to a social category has been found to lead to more stereotypic judgments of the target person, by assimilating the target person to the stereotype (Darley and Gross, 1983; Bodenhausen and Macrae, 1998).

To control for such a halo effect of the stereotype labels, Experiment 3 did not provide category labels. Specifically, stereotype valence and semantic consistency of provided attributes were again manipulated; this time, without providing category information about the stereotypes.

### Method

#### Participants and Design

Participants were *N* = 42 students (32 female, 9 male, 1 unknown) with a mean age of 25 years (*SD* = 6).

#### Materials and Procedure

Experiment 2 was replicated with the only difference that stereotype category labels were not presented (e.g., *Martha is caring, child-loving, affectionate, warm-hearted, empathic*, is a positive, consistent example, consistent with the stereotype midwife). The experiment took about 3 min and was again administered as part of a multi-experiment session.

### Results

**FIGURE 3:**
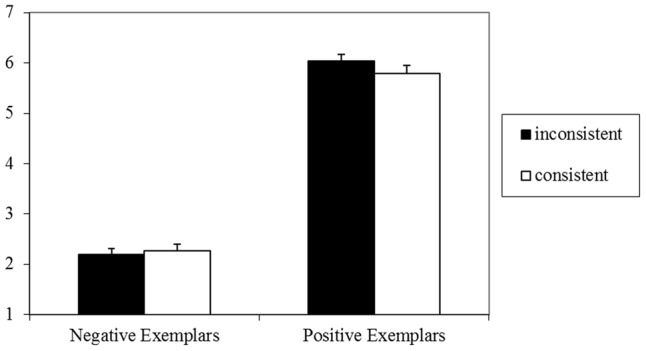
Means and standard errors for evaluation of target persons in Experiment 3 (1 = very negative, 7 = very positive).

The 2 (valence: positive, negative) × 2 (consistency: consistent, inconsistent) repeated measures ANOVA on the preference judgments again found only a main effect of valence, *F*(1,41) = 231.22, *p* < 0.001, $\eta_{p}^{2}$ = 0.85, 90% CI = 0.77–0.89, with more positive evaluations for positive (*M* = 5.78, *SD* = 1.00) compared to negative stereotypes (*M* = 2.02, *SD* = 0.76), and still neither an effect of consistency, *F*(1,41) = 0.12, *p* = 0.72, $\eta_{p}^{2}$ = 0.00, nor an interaction between consistency and valence, *F*(1,41) = 0.72, *p* = 0.40, $\eta_{p}^{2}$ = 0.02. Results are illustrated in **Figure 3**.

### Discussion

Again, apart from a strong valence effect of implied stereotype, no effect of semantic consistency was found, although in the current set-up, no stereotype category labels were shown and thus participants had to rely on piecemeal processing of individuating information of the attributes (e.g., Fiske, 2012). This lack of a consistency effect stands in contrast to earlier findings on coherence-driven preference (Gordon and Holyoak, 1983; Newell and Bright, 2001; for reviews, see Winkielman et al., 2012). For instance, in Topolinski and Strack (2009a, Experiment 3) participants were presented with three semantically consistent (e.g., DEEP FOAM SALT, implying SEA) or inconsistent (e.g., DREAM BALL BOOK, no common associate) words and were asked for their overall evaluation of these word triads, which resulted in more positive evaluations of coherent compared to incoherent word triads. In these experiments, however, the evaluation task was not framed as an impression formation task. Instead, participants incidentally read over words that were not framed as a particular entity or compound (see also Gordon and Holyoak, 1983; Newell and Bright, 2001; Topolinski et al., 2009).

Thus, to realize an experimental set-up as close as possible to earlier consistency-preference findings (Gordon and Holyoak, 1983; Newell and Bright, 2001; Topolinski and Strack, 2009b), Experiment 3 was replicated without an impression formation mind-set. For this, Experiment 4 left out the impression information instruction and first names of the target persons.

## Experiment 4

Experiment 3 was replicated with the sole difference that the attributes were not presented as belonging to a specific target person. Thus, participants read the attributes, without a stereotype label (as in Experiment 3) but also without a first name of a target person (e.g., *caring, child-loving, affectionate, warm-hearted, empathic*). Accordingly, participants were not asked to evaluate a target person but to evaluate a group of words (similar to Topolinski and Strack, 2009a,d).

### Method

#### Participants

*N* = 28 students (22 female, 5 male, 1 unknown) with a mean age of 25 years (*SD* = 4) participated in the study.

#### Materials and Procedure

Experiment 3 was replicated with the following differences. Participants were not presented with target persons but with lists of the attributes from the present stimulus pool of positive and negative stereotype exemplars. Then, they were asked for an evaluation of *this group of words*, instead of an evaluation of a person. The task took about 3 min and was again part of a longer experimental session with other tasks.

### Results

**FIGURE 4:**
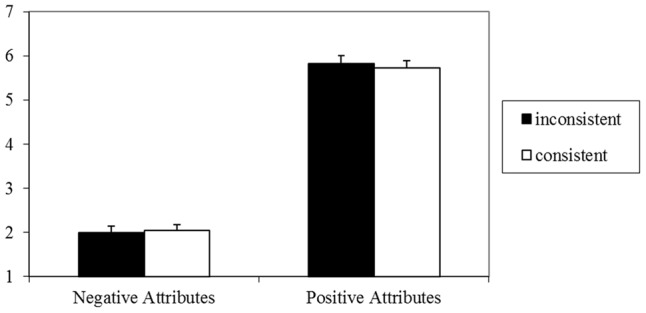
Means and standard errors for evaluation of sets of words in Experiment 4 (1 = very negative, 7 = very positive).

The 2 (valence: positive, negative) × 2 (consistency: consistent, inconsistent) repeated measures ANOVA on the preference ratings for the word groups again found a main effect of valence, *F*(1,27) = 382.00, *p* < 0.001, $\eta_{p}^{2}$ = 0.93, 90% CI = 0.88–0.95, with more positive evaluations of groups of positive attributes (*M* = 6.04, *SD* = 0.76) than of groups of negative attributes (*M* = 1.85, *SD* = 0.59). But still neither an effect of consistency, *F*(1,27) = 0.61, *p* = 0.44, $\eta_{p}^{2}$ = 0.02, nor an interaction between consistency and valence emerged, *F*(1,27) = 1.71, *p* = 0.20, $\eta_{p}^{2}$ = 0.06. Findings are illustrated in **Figure 4**.

### Discussion

Experiment 4 still found no impact of consistency on evaluative judgments, even though only semantically consistent and inconsistent word groups were evaluated. Moreover, the manipulation check in Experiment 2 shows that these word groups do indeed constitute a consistency manipulation. This shows that the lack of consistency effects in the previous experiments is not due to a motivation to make an unbiased judgment elicited by impression formation instructions.

In contrast to earlier successful manipulations (Mendes et al., 2007, who let participants interact directly with the target persons) the present consistency manipulations might have been too subtle. Earlier manipulations used material that was less strongly valenced (e.g., Mendes et al., 2007 used nationalities). As already speculated in the discussion of Experiments 1 and 2, it is possible that the strong stereotype valence affected evaluative judgments so strongly that consistency-driven fluency might be unable to modulate the effects on top of that. Thus, the final experiments use rather neutral stereotypes.

## Experiment 5

To test the influence of consistency on evaluative judgments for moderately valenced stereotypes, Experiment 5 used positive, negative, and neutral stereotypes, that is, stereotypes that are evaluated on average to roughly fall in the middle of the negative–positive continuum. Thus, participants evaluated consistent and inconsistent word groups that were positive, neutral, and negative. A manipulation check, similar to the manipulation check in Experiment 2, tested whether consistency was effectively manipulated. To ensure that a lack of a consistency effect is not due to power issues, we tested a large sample. Additionally, we preregistered the experimental procedure.

### Method

#### Participants

Participants were 228 students (166 female, 50 male, 12 unknown) with a mean age of 23 years (*SD* = 4 years). They were compensated by receiving candy bars for the whole experimental session, which lasted approximately 10 min.

#### Materials and Procedure

The stereotypes and attributes that were identified as relatively neutral in Pilot Study 1 were used (racing driver, butcher, detective, and bookbinder; see **Table 1**) in addition to the positive and negative stereotypes used in Experiments 1–4.

Like in the previous experiments, five attributes that were associated with a certain category were presented in consistent trials. For inconsistent trials, two attributes consistent with the category and three attributes randomly selected from other valence congruent stereotype sets were presented (again, counter-balanced across participants). Due to the random selection, it is possible that some trials in the inconsistent condition did not stay neutral but became valenced by chance because all randomly selected attributes in that trial were positive (negative). This opens up the alternative explanation that a negativity bias might be responsible for any occurring effect (due to more negative novel information for inconsistent compared to consistent targets). However, note that even our neutral stimulus set was rather positive, which renders this interpretation unlikely.

The procedure was identical to Experiment 4, with participants evaluating two word groups for each of the six conditions (negative consistent, negative inconsistent, neutral consistent, neutral inconsistent, positive consistent, positive inconsistent). The experiment took about 5 min and was administered as part of a multi-experiment session.

#### Manipulation Check of Consistency Manipulation

As a manipulation check, *N* = 16 students (six female, three male, seven gender unknown due to technical problems) with a mean age of 27 years (*SD* = 7) were presented with the attributes of the five neutral stereotypes without category labels (like in the manipulation check of Experiment 2) and were asked to rate the coherence of the word group (*How coherent does this group of words seem to you …*, 1 = *completely random*, 7 = *very coherent*). Consistent attribute sets (*M* = 5.47, *SD* = 1.32) were rated as being more coherent than inconsistent attribute sets (*M* = 3.72, *SD* = 1.44), *t*(15) = 3.26, *p* < 0.001, *d_z_* = 0.93, 95% CI = 0.33–1.51. This suggests that semantic consistency was manipulated effectively in the current set-up.

### Results

**FIGURE 5:**
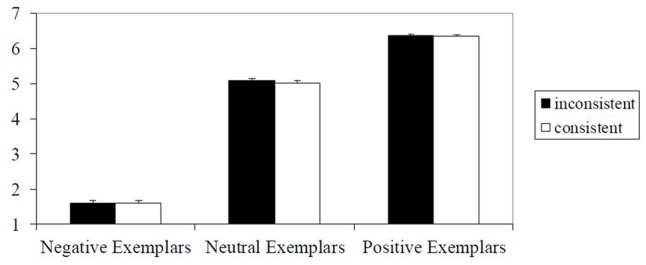
Means and standard errors for evaluation of sets of words in Experiment 5 (1 = very negative, 7 = very positive).

A 2 (consistency: consistent, inconsistent; within) × 3 (valence: negative, neutral, positive; within) repeated measures ANOVA on the preference ratings of the targets found a main effect of valence, *F*(2,226) = 2231.00, *p* < 0.001, $\eta_{p}^{2}$ = 0.95, 90% CI = 0.94–0.96. *Post hoc* tests confirmed that positive sets were evaluated more positively than neutral sets, which, in turn, were evaluated more positively than negative sets (all *p*s < 0.001). There was no main effect of consistency, *F*(1,227) = 0.33, *p* = 0.57, $\eta_{p}^{2}$ < 0.01, and no interaction of consistency and valence, *F*(2,226) = 0.32, *p* = 0.72, $\eta_{p}^{2}$ < 0.01. Results are illustrated in **Figure 5**.

### Discussion

Manipulating the consistency of strongly valenced and neutral stereotype attributes, we again found that consistency of word groups did not influence evaluations. Thus, consistency did not affect preference judgments even for neutral stereotypes, whereas previous research with moderately valenced material did find consistency to influence preferences (Mendes et al., 2007; Topolinski and Strack, 2009a).

However, in Experiment 5, neutral stereotypes were randomly intermixed with strongly valenced stereotypes. Thus, the neutral stereotypes were presented in a strongly valenced context, and this experimental context could still have masked a consistency effect. Accordingly, we performed one more experiment using only neutral stereotypes. Without strong valence, subtle valence effects, such as consistency-induced fluency, should have a better chance of influencing judgments.

## Experiment 6

To test the influence of consistency on evaluative judgments without interference by strongly valenced stereotypes, Experiment 6 used only neutral stereotypes. The experiment was administered at the very beginning of a multi-experiment session to ensure that the valence concept was not primed by other experiments. To ensure that a lack of a consistency effect is not due to power issues, we again tested a large sample.

### Method

#### Participants

Participants were 204 students (133 female, 67 male, 4 chose none of these categories) with a mean age of 22 years (*SD* = 4). The task took about 2 min and was part of a larger battery of unrelated tasks. The whole session took about 35 min, and participants received 5 euro for the whole session.

#### Materials and Procedure

The neutral stereotypes and attributes from Experiment 5 were used (racing driver, butcher, detective, and bookbinder; see **Table 1**).

Like in the prior experiments, five attributes that were associated with a certain category were presented in consistent trials. For inconsistent trials, two attributes consistent with the category and three attributes randomly selected from other neutrally valenced stereotype sets were presented.

As between-subjects manipulation, the attributes were either shown together with a stereotype label and a first name of the target person (as in Experiment 2) or without the stereotype label and without a name (as in Experiments 4 and 5). The procedure was identical to the earlier Experiments 2–5.

### Results

**FIGURE 6:**
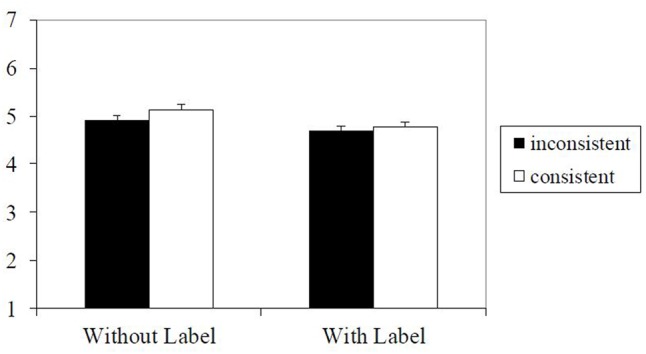
Means and standard errors for evaluation of sets of words/target persons in Experiment 6 (1 = very negative, 7 = very positive).

A 2 (consistency: consistent, inconsistent; within) × 2 (label: with stereotype label, without stereotype label; between) mixed model ANOVA on the preference ratings of the targets found a main effect of label, *F*(1,202) = 7.60, *p* = 0.01, $\eta_{p}^{2}$ = 0.04, 90% CI = 0.01–0.09, with attributes presented without a stereotype label being evaluated more positively than attributes presented with a label. The main effect of consistency was marginally significant, *F*(1,202) = 3.07, *p* = 0.08, $\eta_{p}^{2}$ = 0.015, 90% CI = 0.00–0.06. Consistent stereotypes tended to be evaluated more positively than inconsistent stereotypes. There was no interaction of consistency and label, *F*(1,202) = 0.69, *p* = 0.41, $\eta_{p}^{2}$ < 0.01. Results are illustrated in **Figure 6**.

### Discussion

Manipulating the consistency of attributes of neutral stereotype exemplars, we found that consistent target persons were liked more than inconsistent ones. However, this consistency effect on preference was only marginally significant and very small.

## General Discussion

The present line of research investigated the respective contributions of stimulus-inherent valence and consistency-induced fluency on preference judgments, using social stereotypes. The results are clear-cut for strongly valenced stereotypes. For positive and negative stereotypes, only stimulus valence influenced preferences: Targets from positive stereotypes were generally preferred over targets from negative stereotypes (cf., Anderson, 1962, 1971; Do et al., 2008). Strongly valenced inconsistent attributes shifted evaluations in the direction of their respective valence (Experiment 1). Affect from consistency-driven fluency had no impact (Experiments 2–5), although consistency of the present stimulus material was rigorously manipulated using associations from a normative sample (Pilot Studies), and a manipulation check of consistency was successful (Experiment 2).

The picture is less clear for neutral stereotypes. When neutral stereotypes were presented amongst positive and negative stereotypes (Experiment 5), consistency of the stereotypic attributes did not influence preference judgments. Only when the context stripped of all strong valence cues, did a very small and only marginally significant congruence effect occur for neutral stereotypes (Experiment 6). This is in line with the hypothesis that consistency information is only accessed if no clear valence information is available. In sum, the present research shows that consistency-driven processing fluency influences evaluative judgments only to a very small degree. Its practical relevance can therefore be questioned and will be later in the discussion.

In the current experiments, participants were able to perceive consistency, but apparently, they did not use consistency-induced fluency for their preference judgments unless stereotypes were rather neutral in valence. The manipulation check specifically asked participants to rate consistency (yielding a very large-sized effect), while the experiments assessed participants’ preferences for target persons. It is possible that although participants were perfectly capable of perceiving inconsistency, they simply did not use this information in their evaluative judgment. Valence of character traits might be a more relevant social cue for the evaluation of an interaction partner than consistency of these traits with a preexisting stereotype, leading to the latter information being ignored if valence information was available. In sum, either, even though consistency is recognized, consistency-driven fluency is not experienced amid the much stronger affect resulting from the valenced stereotype information; or consistency-driven fluency is discarded as not useful in preference judgments of valenced target persons.

### The Subtlety of Fluency

From a more general perspective of affective-cognitive undercurrents of preference, the current evidence is highly informative concerning the relative contribution of different sources of affect; specifically the relative contribution of subtle affect due to fluency compared to stimulus-inherent valence. To our knowledge, the current study is the first to orthogonally manipulate stimulus valence and stimulus fluency (see also the mixed evidence on stimulus valence and repetition as fluency induction, Grush, 1976; Dijksterhuis and Smith, 2002; Schellenberg et al., 2008). Although processing fluency has been shown to be a pervasive influence on a variety of judgments (Reber et al., 2004), and semantic consistency has been shown to be a powerful tool to effectively manipulate fluency (Whittlesea, 1993; Topolinski and Strack, 2009a,b,d), in the current studies consistency-driven fluency had only a small impact on preferences for neutral stereotypes (and this only under optimal experimental conditions), but no impact at all on valenced stereotypes.

This clearly shows a boundary condition of fluency effects: when the stimulus itself is strongly valenced and socially meaningful, the affective variations triggered by fluency dynamics (Winkielman and Cacioppo, 2001; Topolinski et al., 2009) are too subtle to influence preference judgments. This is probably due to the fragile experiential status of fluency-variations. Only strong variations of fluency can be experienced consciously, and only under certain conditions of awareness (for perceptual fluency, see Reber et al., 2004; for semantic fluency, see Topolinski and Strack, 2009d). When additional phasic affective input is too strongly valenced, it seems to overshadow the subtle fluency affect.

In a more general sense, the present research examined the influence of a biasing factor in the context of more relevant information. In several domains, biases have been found to be reduced if more relevant information is available. For example, the trustworthiness of a person’s face influences behavior toward that person to a lesser degree when information about the person’s relevant behavior is available (Chang et al., 2010). This is highly adaptive—behavior should be most influenced by the most relevant information. In the present research, we examined the same idea. We examined whether consistency-induced fluency, a biasing factor in many laboratory paradigms, influences judgments about people even when more diagnostic information is available (here, traits). With strongly valenced information, the influence of consistency completely vanished. Even for neutrally valenced information, consistency influenced preference judgments at best to a very small degree. Thus, it seems that consistency-induced fluency’s role in applied settings is small. While this does not invalidate fluency as a cognitive mechanism, its role in applied social settings with rich information available seems minor at best.

### Implications for Additive Accounts of Impression Formation

How information from different stimuli is integrated into evaluative judgments is explained by early additive accounts of information integration (e.g., Anderson, 1962, 1971; for a review, see Massaro and Friedman, 1990). Additive accounts postulate that the overall evaluation of a target person is simply determined by the evaluation of single pieces of information about the target person that are then averaged. In a pioneering study, participants evaluated hypothetical persons on the basis of a random set of three adjectives they learned about the person (e.g., good-natured, bold, humorless). All adjectives had been pre-rated for their valence. The evaluation of the person could be predicted by the arithmetic mean of the evaluation of the individual attributes (Anderson, 1962). Later, additive accounts were refined by a weighing factor, assigning weight to the value of each piece of information depending on its importance or salience (Anderson, 1971), or due to motivational reasons. For instance, negative information was shown to be weighed stronger in evaluative judgments than positive information, because negative information can have a warning function (*negativity bias*, for a review, see Rozin and Royzman, 2001).

Most of the evidence presented here can be accounted for by additive theories of information integration (e.g., Anderson, 1962, 1971). Experiments 1–5 (and particularly Experiment 1 confounding information valence with consistency) showed that the higher the number of positive attributes presented with the target person, the more positive was the evaluation of the target person.

In contrast, in Experiment 6 evaluative judgments differed contingent on consistency, even though stimulus valence was constant between consistent and inconsistent target persons. Additive accounts of information integration have difficulties in explaining the differences in the evaluation of consistent compared to inconsistent neutral target persons. As consistent and inconsistent stimuli were sampled from the same pool of attributes, their average valence could not differ. Thus, the observed small difference in the evaluative judgment of consistent and inconsistent target persons cannot stem from differences in stimulus valence. The only difference between the two conditions was the semantic consistency of the attributes presented with the target person, which triggered cognitive feelings of ease above and beyond stimulus valence (cf., Schwarz, 1990, 2002, 2012).

In sum, the present Experiments 1–5 support additive accounts of information integration in an impression formation task using strongly valenced stereotypes. However, the results of Experiment 6 cannot be explained fully by additive information integration. Here, consistency-triggered fluency seem to have influenced preference judgments.

### Implications for Stereotype Change

Stereotypes have been shown to be pervasive (Devine, 1989; Devine and Sharp, 2009) and hard to change (Weber and Crocker, 1983). Because activation and application of stereotypes can have negative effects (Stangor, 2009), the attempt to reduce the application of stereotypes or to even change stereotypic representations is one of the oldest and most prominent research aims in social psychology (Allport, 1954; Bodenhausen et al., 2009). One of the factors frequently discussed as a possible means to stereotype change is the confrontation with stereotype-inconsistent behavior or atypical members of stereotyped groups (Allport, 1954; Weber and Crocker, 1983; Tausch and Hewstone, 2010).

Will an individual’s stereotype about hooligans be altered by encountering a child-loving hooligan? This is exactly what theories of intergroup contact would predict and what empirical research on stereotype change has found (Allport, 1954; Kunda and Oleson, 1995, 1997; Brown and Hewstone, 2005; for a review, see Pettigrew and Tropp, 2006; but see for boundary conditions, e.g., Weber and Crocker, 1983; Fyock and Stangor, 1994; Trope and Thompson, 1997). As changing stereotypes on a large scale promises the solution to many intergroup conflicts, it has received immense attention over the last 100 years (Stangor, 2009). However, a widely ignored factor in this equation is the stereotype-inconsistent individual. What does the child-loving hooligan gain or suffer from displaying stereotype-inconsistent behavior? The answer that can be derived from the present findings is twofold. For strongly valenced stereotypes about the group, inconsistent information only had a strong effect on evaluative judgments of the individual when this information was affectively mismatching (Experiment 1).

However, the situation was different when stereotypes were not strongly valenced in the first place. Stereotype-inconsistent individuals from neutral groups tended to be evaluated less positively than stereotype-consistent individuals. For example, a literate butcher tended to be evaluated less positively than a strong butcher (Experiment 6). This shows that the effects of stereotype-inconsistent information that can possibly help to alter stereotypic beliefs about the stereotyped group as a whole can be at the expense of the inconsistent individual (cf., Mendes et al., 2007; Phelan et al., 2008; Rudman et al., 2012). However, as the present inconsistency effects were small and occurred only for neutral stereotypes and under certain experimental conditions, negative consequences for the inconsistent individual should not be overestimated and are likely to be irrelevant in enriched social interactions.

## Conclusion

The present line of experiments showed that evaluative judgments in stereotype disconfirmation are mainly driven by stimulus valence. Inconsistency-driven processing fluency did only influence evaluative judgments in the absence of strongly valenced stimuli, and only to a very small degree. This shows that while effects of processing dynamics are intriguing and give us further insight into the functioning of human cognition, their relative impact on evaluative judgments in a social context seems to be small.

## Ethics Statement

This study was carried out in accordance with the recommendations of the German Research Foundation (DFG) of guidelines, with written informed consent from all subjects. All subjects gave written informed consent in accordance with the Declaration of Helsinki.

## Author Contributions

LS conducted the research presented in this manuscript with contributions to the conception and design by ST and FS. LS and AK analyzed the data. BL contributed to the interpretation of the results and their discussion. LS, ST, and AK wrote the first draft of the manuscript. BL and FS provided important theoretical input and critically revised the manuscript. All authors give their approval of the version of the manuscript to be published and agree to be accountable for all aspects of the work.

## Conflict of Interest Statement

The authors declare that the research was conducted in the absence of any commercial or financial relationships that could be construed as a potential conflict of interest. The reviewer ED and handling Editor declared their shared affiliation.

## Footnotes

1 https://osf.io/twgj5/?view_only=fe3bc69e75c6423a9896bde03eccda31

2 https://osf.io/xsgm2/?view_only=cbd7f18ddcbe4d8bb5b37f4fb81e8a32
